# Application of Theory of Quantum Instruments to Psychology: Combination of Question Order Effect with Response Replicability Effect

**DOI:** 10.3390/e22010037

**Published:** 2019-12-26

**Authors:** Masanao Ozawa, Andrei Khrennikov

**Affiliations:** 1 Graduate School of Informatics, Nagoya University, Chikusa-ku, Nagoya 464-8601, Japan; ozawa@is.nagoya-u.ac.jp; 2 College of Engineering, Chubu University, 1200 Matsumoto-cho, Kasugai 487-8501, Japan; 3 International Center for Mathematical Modeling in Physics and Cognitive Sciences, Linnaeus University, SE 351 95 Växjö, Sweden

**Keywords:** quantum-like models, psychology, decision making, social science, quantum instruments, order effect, response replicability effect, non-atomic quantum instruments

## Abstract

Recently, quantum formalism started to be actively used outside of quantum physics: in psychology, decision-making, economics, finances, and social science. Human psychological behavior is characterized by a few basic effects; one of them is the question order effect (QOE). This effect was successfully modeled (Busemeyer–Wang) by representing questions *A* and *B* by Hermitian observables and mental-state transformations (back action of answering) by orthogonal projectors. However, then it was demonstrated that such representation cannot be combined with another psychological effect, known as the response replicability effect (RRE). Later, this no-go result was generalized to representation of questions and state transformations by quantum instruments of the atomic type. In light of these results, the possibility of using quantum formalism in psychology was questioned. In this paper, we show that, nevertheless, the combination of the QOE and RRE can be modeled within quantum formalism, in the framework of theory of non-atomic quantum instruments.

## 1. Introduction

Recent years have been characterized by a real explosion of interest to applications of quantum measurement theory and other parts of quantum theory outside of physics, especially in psychology, decision-making, economics, finances, and social science, as well as in genetics and molecular biology. (The work of [1,2,3,4,5,6,7,8,9,10,11,12,13,14,15,16,17,18,19,20,21,22,23,24,25,26,27,28,29,30,31,32,33,34,35,36,37,38,39,40,41,42,43,44,45,46,47,48,49] for some representative papers.) We call the corresponding models quantum-like. They are not about the genuine physical processes in the human brain (cf. Penrose [50], Umezawa [51], Hameroff [52], and Vitiello [53,54]). In the next section, we shall discuss the basics of quantum-like modeling. The introduction section is lengthy because the paper is directed toward multidisciplinary readership. Thus, such an introduction is important to introduce the basic concepts and problems from psychology, decision-making, and quantum theory. In particular, the paper is based on theory of quantum instruments, which is practically unknown for non-physicists.

### 1.1. Quantum-Like Modeling

Quantum-like models [3] reflect those specialties of cognitive information processing which match well with quantum formalism. We emphasize that the strategy of quantum-like modeling does not assume that the whole body of quantum theory should be explored. By doing such modeling, one has to take into account that, in contrast to quantum physics, it would not be natural to claim completeness of the quantum description, i.e., impossibility to create a finer description of processes than given by quantum states and observables. The quantum-like representation of mental states and (self-)observations is operational. It does not provide the complete picture of cognitive processes, including functioning of neuronal networks in the brain. In short, “mental hidden variables” do exist and one has to search the possibility to peacefully combine their existence with quantum theory. The work of [55,56,57,58] covers classical probability representation for quantum mechanics and the tomographic approach, while that of [15,18,59] includes contextual representation Further examples are [60]’s work in classical conditional probability viewpoint on quantum probability, as well as [48]’s uncertainty in the generation of action potential by neurons to the quantum information representation of information in the brain.

Now, we turn to the important foundational issue which is common for applications of quantum theory both in physics and psychology. Quantum theory was not defined from physically natural principles (in contrast, to say special relativity theory, see, e.g., Zeilinger [61] for discussion). The validity of the quantum approach to physics was confirmed by thousands of successful experiments and technological inventions. However, one can still have doubts that the whole body of micro-physics can be covered by quantum formalism (e.g., experiments of the group of Weihs [62]). In psychology, the situation is worse. The doubts in adequacy of quantum theory to psychological (and social) phenomena are stronger.

These doubts were culminated in [24], where the applicability of quantum measurement theory was questioned on the basis of a simple and natural example, the Clinton–Gore opinion pool [63]. In this opinion-polling experiment, people were asked one question at a time, e.g.,

- *A* = “Is Bill Clinton honest and trustworthy?”
- *B* = “Is Al Gore honest and trustworthy?”

Two sequential probability distributions were calculated on the basis of the experimental statistical data, $p_{AB}$ and $p_{BA}$ (first question *A* and then question *B* and vice versa).

### 1.2. Question Order Effect

The statistical data from this experiment was widely used to demonstrate the QOE, dependent on the (sequential) joint probability distribution on the questions’ order: $p_{AB}\neq p_{BA}.$ We recall that QOE served as one of the basic motivations for applying the quantum calculus of probability to psychological phenomena Wang and Busemeyer [21]. Roughly speaking, Wang and Busemeyer [21] reasoned that QOE can be modeled by using non-commutativity of incompatible quantum observables A,B, Hermitian operators. One observable represents the question about Clinton and another about Gore. So, from this viewpoint, QOE is identical incompatibility of observables.

### 1.3. Response Replicability

However, a strong objection to this strategy was presented in [24]. This objection is related to the notion of response replicability, which plays the important role in quantum physics. Response replicability is also the common feature of human behavior. Suppose that Alice is asked some question *A*, and she replies, e.g., “yes”. If immediately after answering she is asked this question again, then she replies “yes” with probability one. We call this property A−A *response replicability*. In physics, instead of questions, one considers physical observables. The A−A response replicability is the common feature of basic quantum experiments, and it is expressed by *the projection postulate*.

This is a good place to stress that the projection postulate describes not measurement by itself, i.e., the statistics of outcomes, but the measurement’s back action modifying system’s state. Thus, in principle, even by assuming that observable *A* is mathematically represented by a projector, one need not assume the validity of the projection postulate. We also remark that this postulate is one of the most questionable postulates of quantum mechanics; see, e.g., [27] for the corresponding discussion in quantum-like modeling).

The Clinton–Gore opinion poll, as well as typical decision-making experiments, satisfy A−A response replicability. However, in psycho-physical experiments, A−A response replicability can be easily violated.

Human decision-making has another distinguishing feature, which can be called A−B−A *response replicability*. Suppose that, after answering the *A*-question with say the “yes”-answer, Alice is asked another question B. She replies to it with some answer. And then she is asked *A* again. In the social opinion pools and other natural decision-making experiments, Alice definitely repeats her original answer to A, “yes”. This is A−B−A response replicability. The combination of A−A with A−B−A and B−A−B response replicability is called the response replicability effect (RRE).

### 1.4. Is It Possible to Combine QOE and RRE in Quantum Formalism?

As was pointed out in [24], by using the calculus of Hermitian observables and the projection postulate, it is impossible to combine RRE with QOE. In short, to generate QOE Hermitian operators A,B should be non-commutative, but the latter destroys A−B−A response replicability of A. This is the very strong objection to the use of quantum formalism in psychology.

However, even in physics, as was already mentioned, general applicability of the projection postulate was questioned. This led to generalization of quantum measurement theory originally instituted by von Neumann [64]. Today, the most general measurement theory is based on theory of quantum instruments; see Davies and Levis [65,66] and Ozawa [67,68,69,70] for basics of this theory (see also [71,72,73,74,75,76,77,78] for further developments). This theory essentially extends the class of possible state transformation which can be generated by measurement back action, as well as the class of possible operators representing observables. The latter are given by positive operator valued measures (POVMs). We point out that, recently, POVMs started to be used, but very slowly, even in psychology (Khrennikov [15], Basieva–Khrennikov [27,79], Khrennikova and Haven [39]).

We mention that Basieva–Khrennikov [79] studied the problem of combining QOE and RRE within the simplest class of quantum instruments of non-projection type, so-called atomic instruments (Section 2.2). We remark that such instruments are widely applied in quantum information theory. It was shown that it is impossible to describe the combination of QOE and RRE with atomic instruments This result made the use of quantum-like models in cognition, psychology, decision-making, and social science even more questionable.

In the present paper, we show that theory of quantum instruments provides a simple solution of the combination of QOE and RRE effects in psychology. Thus, the black cloud on the blue sky of quantum-like modeling of cognition disappeared. The solution, although very natural after it was found, is really nontrivial because the quantum instruments that will be used for this problem are not so common in quantum physical applications.

The main lesson of this problem study (starting with [21,24]) is that the essence of QOE is not in the structure of observables, but in the structure of the state transformation generated by measurements’ back action. QOE is not about the joint measurement and incompatibility (non-commutativity) of observables, but it is about sequential measurement of observables and sequential (mental-)state update.

Quantum instruments that will be used to combine QOE and RRE correspond to measurement of commuting observables A,B. The latter is in the striking contrast with the original idea of Busemeyer and Wang that quantum-like modeling of (OE) should be based on the use of non-commutative observables-operators. (The latter statement should not be considered as critique of the works of Busemeyer and Wang; without these works, we would never understand the tricky structure of quantum-like modeling of the combination of (OE) and RRE.).

Moreover, it is possible to prove that, under natural mathematical restriction, QOE and RRE can be jointly modeled with only the aid of quantum instruments for commuting observables.

## 2. Representation of (Self-)Measurement Back Action on System’s State by Quantum Instruments

Denote by H a complex Hilbert space. For simplicity, we assume that it is finite dimensional. Pure states of a system *S* are given by normalized vectors of H and mixed states by density operators. The space of density operators is denoted by S(H).

### 2.1. Von Neumann–Lüders Instruments

First, consider the original quantum formalism (von Neumann, Dirac, Lüders). Here, physical observable *a* is represented by a Hermitian operator A. We consider only operators with discrete spectra:

$$A=\sum_{x}x\;E^{A}\left(x\right),$$

where $E^{A}\left(x\right)$ is the projector onto the subspace of *H* corresponding to the eigenvalue x. Suppose that system’s state is mathematically represented by a density operator ρ. Then, the probability to get the answer *x* is given by the Born rule

(1) $$\mathrm{Pr}\left\{A=x∥\rho \right\}=\mathrm{Tr}\left[E^{A}\left(x\right)\rho \right]=\mathrm{Tr}\left[E^{A}\left(x\right)\rho E^{A}\left(x\right)\right],$$

and the post-measurement state is obtained via the state-transformation:

(2) $$\rho \to \rho_{x}=\frac{E^{A}\left(x\right)\rho E^{A}\left(x\right)}{TrE^{A}\left(x\right)\rho E^{A}\left(x\right)}.$$

Here, the observable-operator *A* (its spectral decomposition) uniquely determines the back action state transformations $I_{A}\left(x\right)$ for outcomes *x*:

(3) $$\rho \to I_{A}\left(x\right)\rho =E^{A}\left(x\right)\rho E^{A}\left(x\right).$$

Such a strategy is very convenient, since one can proceed with Hermitian operator *A* and forget about the associated map $x\to I_{A}\left(x\right)$ to the space of transformations in the space of density operators S(H). Such transformations are called *superoperators.* The map $x\to I_{A}\left(x\right)$, given by Equation (3), is the simplest—but very important—example of quantum instrument.

### 2.2. Atomic Instruments

Let *A* be a Hermitian operator on H since it is now treated as just a symbolic representation of some quantum observable, denoted by the same symbol. Thus, in contrast to Section 2.1, we do not couple the operator *A* to the projection measurement scheme, given by the spectral decomposition of *A*. The crucial point is that the *a*-observable can be measured in many ways. Formally, such measurements schemes are represented by quantum instruments. Now, we consider the simplest class of quantum instruments, so-called atomic instruments.

Consider now a POVM {F(x)}. Here, F(x) is a positive Hermitian operator (called effect) for each *x*, and the normalization condition $\sum_{x}F\left(x\right)=I$ holds, where *I* is the unit operator. Then,

(4) Pr{x=x∥ρ}=Tr[F(x)ρ]

defines the probability distribution of the outcome x of the measurement of POVM F(x). If the POVM F(x) describes the observable *A*, we have

(5) $$\mathrm{Tr}\left[F\left(x\right)\rho \right]=\mathrm{Tr}\left[E^{A}\left(x\right)\rho \right]$$

for any state ρ, so that $F\left(x\right)=E^{A}\left(x\right)$ for all *x*.

What about the back action transformations? Typically it is assumed that effects are represented concretely in the form

(6) $$F\left(x\right)=V{\left(x\right)}^{☆}V\left(x\right),$$

where V(x) is a linear operator on H. Hence, the normalization condition has the form $\sum_{x}V{\left(x\right)}^{☆}V\left(x\right)=I.$ Then, the Born rule can be written similarly to Equation (1):

(7) $$\mathrm{Pr}\left\{x=x∥\rho \right\}=\mathrm{Tr}[V\left(x\right)\rho V{\left(x\right)}^{☆}].$$

It is assumed that the post-measurement state transformation is based on the map:

(8) $$\rho \to I_{A}\left(x\right)\rho =V\left(x\right)\rho V^{☆}\left(x\right),$$

so

(9) $$\rho \to \rho_{x}=\frac{I_{A}\left(x\right)\rho }{TrI_{A}\left(x\right)\rho }.$$

The map $x\to I_{A}\left(x\right)$, given by Equation (8), is atomic quantum instrument. For such instruments, one can speak solely about the POVM-observable and its representation in the form (6).

We remark that the Born rule (7) can be written in the form

(10) $$\mathrm{Pr}\left\{A=x∥\rho \right\}=\mathrm{Tr}\;[I_{A}\left(x\right)\rho ].$$

### 2.3. General Notion of Quantum Instrument

The space of linear Hermitian operators in H is linear space over real numbers. We consider linear operators acting in it *superoperators*. A superoperator is called positive if it maps the set of positive semi-definite operators into itself. We remark that, for each x, $I_{A}\left(x\right)$, given by Equation (8), can be considered as linear positive map in $L_{s}\left(H\right).$

Now, generally any map $x\to I_{A}\left(x\right),$ wherein for each *x* the map $I_{A}\left(x\right)$ is a positive superoperator, is called a *quantum instrument*. Here, index *A* denotes the observable coupled to this instrument. The probabilities of *A*-outcomes are given by Born’s rule in form (10) and the state-update by transformation (9).

## 3. Quantum Instruments from the Scheme of Indirect Measurements

Formally, an indirect measurement model for a Hilbert space H, introduced in [67] as a “(general) measuring process”, is a quadruple $\left(K,\xi ,U,M_{A}\right)$ consisting of a Hilbert space K, a unit vector ξ, a unitary operator *U* on H⊗K, and a Hermitian operator $M_{A}$ on K. By this measurement model $\left(K,\xi ,U,M_{A}\right)$, the Hilbert space K describes the probe system $\tilde{S}$, the unitary operator *U* describes the time-evolution of the composite system $S+\tilde{S}$, the unit vector ξ describes the initial state of the probe $\tilde{S}$, and the operator $M_{A}$ describes the meter observable in the probe $\tilde{S}$ to be measured directly just after the interaction. Then, the output probability distribution Pr{A=x∥ρ} in the system state ρ∈S(H) is given by

(11) $$\mathrm{Pr}\left\{A=x∥\rho \right\}=\mathrm{Tr}[\left(I⊗E^{M_{A}}\left(x\right)\right)U(\rho ⊗|\xi \rangle \langle \xi |)U^{†}],$$

where $E^{M_{A}}\left(x\right)$ is the spectral projection of $M_{A}$ for the eigenvalue x.

The change of the state ρ of the system *S* caused by the measurement for the outcome A=x is represented with the aid of the map $I_{A}\left(x\right)$ in the space of density operators defined as

(12) $$I_{A}\left(x\right)\rho ={\mathrm{Tr}}_{K}\left[\left(I⊗E^{M_{A}}\left(x\right)\right)U(\rho ⊗|\xi \rangle \langle \xi |)U^{†}\right],$$

where ${\mathrm{Tr}}_{K}$ is the partial trace over K. The map $x\mapsto I_{A}\left(x\right)$ is a quantum instrument.

We remark that under a natural restriction—so-called complete positivity—any quantum instrument can be represented via the indirect measurement model [67].

## 4. Constructions of Instruments Describing QOE and RRE Effects

### 4.1. Observables A and B

Let *A* and *B* be projections on a Hilbert space H. They correspond to questions labeled *A* and B, respectively. The eigenvalue 1 means the answer “yes” to the questions, and the eigenvalue 0 means the answer “no” to the questions. We consider the case where projections *A* and *B* commute. In particular, we let $H={\{|00\rangle ,|01\rangle ,|10\rangle ,|11\rangle \}}^{⊥⊥}$, where ${}^{⊥}$ stands for the orthogonal complement in H, so that $S^{⊥⊥}$ stands for the subspace spanned by a subset *S* of H. Denote by $I_{1}$ and $I_{2}$ the identity operators on the first and the second qubit in H, respectively. We let $A=\left|1\rangle \langle 1\right|⊗I_{2}$, and $B=I_{1}⊗\left|1\rangle \langle 1\right|$.

### 4.2. Instrument $I_{A}$ Measuring A

We construct an instrument $I_{A}$ measuring *A* as follows. The instrument $I_{A}$ carries out a measurement of *A* by a measuring interaction between the object S described by the state space H and the probe P described by the state space $K={\{|00\rangle ,|01\rangle ,|10\rangle ,|11\rangle \}}^{⊥⊥}$; denote by $I_{3}$ and $I_{4}$ the identity operators on the first and the second qubit in K. The time evolution of the composite system S+P during the measuring interaction is described by a unitary operator $U_{A}$ on H⊗K satisfying (it is easy to see that there exists at least one unitary operator $U_{A}$ on H⊗K satisfying the given relations, since the relations define a one-to-one correspondence between two subsets of the orthonormal basis {|α,β,γ,δ〉∣α,β,γ,δ=0,1} of H⊗K)

(13) $$\begin{array}{cc} U_{A}:|00\rangle |00\rangle & \mapsto |01\rangle |00\rangle , \end{array}$$

(14) $$\begin{array}{cc} U_{A}:|01\rangle |00\rangle & \mapsto |01\rangle |01\rangle , \end{array}$$

(15) $$\begin{array}{cc} U_{A}:|10\rangle |00\rangle & \mapsto |10\rangle |10\rangle , \end{array}$$

(16) $$\begin{array}{cc} U_{A}:|11\rangle |00\rangle & \mapsto |10\rangle |11\rangle , \end{array}$$

and the outcome of the measurement is obtained by measuring the meter observable

(17) $$M_{A}=\left|1\rangle \langle 1\right|⊗I_{4}$$

of the probe P, i.e., measuring the first qubit of K. Note that both *A* and $M_{A}$ have the same spectrum {0,1}, and they are projections. Suppose that the probe P is prepared in the state |00〉 just before the measuring interaction, the measuring process described by $(K,|00\rangle ,U_{A},M_{A})$ defines the instrument $I_{A}$ by

(18) $$\begin{array}{cc} I_{A}\left(0\right)\rho & ={\mathrm{Tr}}_{K}\left[\left(I_{H}⊗M_{A}^{⊥}\right)U_{A}(\rho ⊗|00\rangle \langle 00|)U_{A}^{†}\left(I_{H}⊗M_{A}^{⊥}\right)\right], \end{array}$$

(19) $$\begin{array}{cc} I_{A}\left(1\right)\rho & ={\mathrm{Tr}}_{K}\left[\left(I_{H}⊗M_{A}\right)U_{A}(\rho ⊗|00\rangle \langle 00|)U_{A}^{†}\left(I_{H}⊗M_{A}\right)\right] \end{array}$$

for any density operator ρ on H, where ${\mathrm{Tr}}_{K}$ stands for the partial trace over K, where $I_{H}$ stands for the identity operator on H, i.e., $I_{H}=I_{1}⊗I_{2}$, and where $M_{A}^{⊥}$ is the orthogonal complement of the projection $M_{A}$, i.e., $M_{A}^{⊥}=I_{K}-M_{A}$, where $I_{K}=I_{3}⊗I_{4}$.

The measurement described by the instrument $I_{A}$ determines the probability distribution Pr{x=x|ρ} of the outcome x of the measurement, where x=0,1, and the state change $\rho \mapsto \rho_{\left\{x=x\right\}}$ caused by the measurement by

(20) $$\begin{array}{cc} \mathrm{Pr}\{x=x|\rho \} & =\mathrm{Tr}[I_{A}\left(x\right)\rho ], \end{array}$$

(21) $$\begin{array}{cc} \rho \mapsto \rho_{\left\{x=x\right\}} & =\frac{I_{A}\left(x\right)\rho }{\mathrm{Tr}[I_{A}\left(x\right)\rho ]}. \end{array}$$

Suppose that the object state |ψ〉 just before the measurement is arbitrarily given, i.e., $|\psi \rangle =\sum_{\alpha ,\beta }c_{\alpha ,\beta }|\alpha ,\beta \rangle$. Then, by the Born formula, we have

(22) $$\begin{array}{cc} \mathrm{Pr}\{A=0||\psi \rangle \} & ={∥A^{⊥}|\psi \rangle ∥}^{2}={\left|c_{00}\right|}^{2}+{\left|c_{01}\right|}^{2}, \end{array}$$

(23) $$\begin{array}{cc} \mathrm{Pr}\{A=1||\psi \rangle \} & ={∥A|\psi \rangle ∥}^{2}={\left|c_{10}\right|}^{2}+{\left|c_{11}\right|}^{2}. \end{array}$$

By the linearity of $U_{A}$, it follows from Equations (13)–(16) that

(24) $$\begin{array}{c} U_{A}:|\psi \rangle |00\rangle \mapsto c_{0,0}|01\rangle |00\rangle +c_{0,1}|01\rangle |01\rangle +c_{1,0}|10\rangle |10\rangle +c_{1,1}|10\rangle |11\rangle , \end{array}$$

and we have

$$\begin{array}{cc} \mathrm{Pr}\{M_{A}=0|\;U_{A}|\psi \rangle |00\rangle \} & ={∥\left(I_{H}⊗M_{A}^{⊥}\right)U_{A}|\psi \rangle |00\rangle ∥}^{2}=|c_{00}{|}^{2}+{\left|c_{01}\right|}^{2},\\ \mathrm{Pr}\{M_{A}=1|\;U_{A}|\psi \rangle |00\rangle \} & ={∥\left(I_{H}⊗M_{A}\right)U_{A}|\psi \rangle |00\rangle ∥}^{2}=|c_{10}{|}^{2}+{\left|c_{11}\right|}^{2}. \end{array}$$

This shows

$$\begin{array}{cc} \mathrm{Pr}\{M_{A}=0|\;U_{A}|\psi \rangle |00\rangle \} & =\mathrm{Pr}\{A=0|\;|\psi \rangle \},\\ \mathrm{Pr}\{M_{A}=1|\;U_{A}|\psi \rangle |00\rangle \} & =\mathrm{Pr}\{A=1|\;|\psi \rangle \} \end{array}$$

for any state |ψ〉 in H. It follows that the instrument $I_{A}$ measures the observable *A*, i.e.,

$$\begin{array}{cc} \mathrm{Pr}\{x=0|\rho \} & =\mathrm{Tr}\left[I_{A}\left(0\right)\rho \right]=\mathrm{Tr}\left[A^{⊥}\rho \right],\\ \mathrm{Pr}\{x=1|\rho \} & =\mathrm{Tr}[I_{A}\left(1\right)\rho ]=\mathrm{Tr}\left[A\rho \right] \end{array}$$

for any density operator ρ on H.

To derive the state change caused by the measurement, suppose that the observer measures the meter observable $M_{A}$ just after the measuring interaction. Then, by Equation (24), we have

$$\left(I_{H}⊗M_{A}^{⊥}\right)U_{A}|\psi \rangle |00\rangle =c_{0,0}|01\rangle |00\rangle +c_{0,1}|01\rangle |01\rangle ,$$

and by Equation (18), we have

$$\begin{array}{cc} I_{A}\left(0\right)|\psi \rangle \langle \psi | & ={\left|c_{0,0}\right|}^{2}\left|01\rangle \langle 01|+\right|c_{0,1}{|}^{2}\left|01\rangle \langle 01\right|\\ & =|01\rangle \langle 00|\psi \rangle \langle \psi |00\rangle \langle 01|+|01\rangle \langle 01|\psi \rangle \langle \psi |01\rangle \langle 01|\\ & =\mathrm{Tr}[A^{⊥}|\psi \rangle \langle \psi |]|01\rangle \langle 01|. \end{array}$$

By the linearity of $I_{A}\left(0\right)$, we conclude

$$\begin{array}{c} I_{A}\left(0\right)\rho =\mathrm{Tr}\left[A^{⊥}\rho \right]\left|01\rangle \langle 01\right| \end{array}$$

for any density operator ρ on H. Similarly, we have

$$\begin{array}{cc} I_{A}\left(1\right)\rho & =\mathrm{Tr}[A\rho ]|10\rangle \langle 10|. \end{array}$$

Thus, the state change caused by the measurement is given by

$$\begin{array}{cc} \rho \mapsto \rho_{\left\{x=0\right\}} & =\frac{I_{A}\left(0\right)\rho }{\mathrm{Tr}[I_{A}\left(0\right)\rho ]}=|01\rangle \langle 01|,\\ \rho \mapsto \rho_{\left\{x=1\right\}} & =\frac{I_{A}\left(1\right)\rho }{\mathrm{Tr}[I_{A}\left(1\right)\rho ]}=|10\rangle \langle 10|. \end{array}$$

### 4.3. Instrument $I_{B}$ Measuring B

The instrument $I_{B}$ is constructed with the same probe system P prepared in the state |00〉. The unitary operator $U_{B}$ on H⊗K, describing the time evolution of the composite system S+P during the measuring interaction, is supposed to satisfy

(25) $$\begin{array}{cc} U_{B}:|00\rangle |00\rangle & \mapsto |10\rangle |00\rangle , \end{array}$$

(26) $$\begin{array}{cc} U_{B}:|01\rangle |00\rangle & \mapsto |01\rangle |01\rangle , \end{array}$$

(27) $$\begin{array}{cc} U_{B}:|10\rangle |00\rangle & \mapsto |10\rangle |10\rangle , \end{array}$$

(28) $$\begin{array}{cc} U_{B}:|11\rangle |00\rangle & \mapsto |01\rangle |11\rangle , \end{array}$$

and the meter observable on K is given by

(29) $$M_{B}=I_{3}⊗|1\rangle \langle 1|.$$

Then, the instrument $I_{B}$ of this measuring process $(K,|00\rangle ,U_{B},M_{B})$ is defined by

(30) $$\begin{array}{cc} I_{B}\left(0\right)\rho & ={\mathrm{Tr}}_{K}\left[\left(I_{H}⊗M_{B}^{⊥}\right)U_{B}(\rho ⊗|00\rangle \langle 00|)U_{B}^{†}\left(I_{H}⊗M_{B}^{⊥}\right)\right], \end{array}$$

(31) $$\begin{array}{cc} I_{B}\left(1\right)\rho & ={\mathrm{Tr}}_{K}\left[\left(I_{H}⊗M_{B}\right)U_{B}(\rho ⊗|00\rangle \langle 00|)U_{B}^{†}\left(I_{H}⊗M_{B}\right)\right] \end{array}$$

for any density operator ρ on H. Then, we have

$$\begin{array}{cc} I_{B}\left(0\right)\rho & =\mathrm{Tr}\left[B^{⊥}\rho \right]|10\rangle \langle 10|,\\ I_{B}\left(1\right)\rho & =\mathrm{Tr}[B\rho ]|01\rangle \langle 01| \end{array}$$

for any density operator ρ on H. Thus, the measurement statistics for the outcome y of the instrument $I_{B}$ are given by

$$\begin{array}{cc} \mathrm{Pr}\{y=0|\rho \} & =\mathrm{Tr}\left[I_{B}\left(0\right)\rho \right]=\mathrm{Tr}\left[B^{⊥}\rho \right],\\ \mathrm{Pr}\{y=1|\rho \} & =\mathrm{Tr}[I_{B}\left(1\right)\rho ]=\mathrm{Tr}\left[B\rho \right],\\ \rho \mapsto \rho_{\left\{y=0\right\}} & =\frac{I_{B}\left(0\right)\rho }{\mathrm{Tr}[I_{B}\left(0\right)\rho ]}=\left|10\rangle \langle 10\right|,\\ \rho \mapsto \rho_{\left\{y=1\right\}} & =\frac{I_{B}\left(1\right)\rho }{\mathrm{Tr}[I_{B}\left(1\right)\rho ]}=\left|01\rangle \langle 01\right| \end{array}$$

for any density operator ρ on H.

## 5. $I_{A}$ and $I_{B}$ Have Both RRE and QOE

In what follows, we shall show that instruments $I_{A}$ and $I_{B}$ have both RRE and QOE.

**Theorem** **1.** *The instruments $I_{A}$ and $I_{B}$ have the Response Replicability Effect; namely, they satisfy the following relations.*

*1.*  *$\sum_{x}\mathrm{Tr}\left[I_{A}\left(x\right)I_{A}\left(x\right)\rho \right]=1$ for any density operator ρ.*
*2.*  *$\sum_{y}\mathrm{Tr}\left[I_{B}\left(y\right)I_{B}\left(y\right)\rho \right]=1$ for any density operator ρ.*
*3.*  *$\sum_{x,y}\mathrm{Tr}\left[I_{A}\left(x\right)I_{B}\left(y\right)I_{A}\left(x\right)\rho \right]=1$ for any density operator ρ.*
*4.*  *$\sum_{x,y}\mathrm{Tr}\left[I_{B}\left(y\right)I_{A}\left(x\right)I_{B}\left(y\right)\rho \right]=1$ for any density operator ρ.*

**Proof.**  For any density operator ρ we have

$$\begin{array}{cc} I_{A}\left(0\right)I_{A}\left(0\right)\rho & =\mathrm{Tr}\left[A^{⊥}\rho \right]I_{A}\left(0\right)\left|01\rangle \langle 01\right|\\ & =\mathrm{Tr}\left[A^{⊥}\rho \right]\mathrm{Tr}[A^{⊥}|01\rangle \langle 01||01\rangle \langle 01|\\ & =\mathrm{Tr}\left[A^{⊥}\rho \right]|01\rangle \langle 01|,\\ I_{A}\left(1\right)I_{A}\left(1\right)\rho & =I_{A}\left(1\right)\mathrm{Tr}\left[A\rho \right]|10\rangle \langle 10|\\ & =\mathrm{Tr}[A\rho ]\mathrm{Tr}[A|10\rangle \langle 10||10\rangle \langle 10|\\ & =\mathrm{Tr}[A\rho ]|10\rangle \langle 10|,\\ \sum_{x}\mathrm{Tr}\left[I_{A}\left(x\right)I_{A}\left(x\right)\rho \right] & =\mathrm{Tr}\left[A^{⊥}\rho \right]+\mathrm{Tr}\left[A\rho \right]\\ & =1. \end{array}$$

Thus, relation (i) follows, and relation (ii) similarly follows. For any density operator ρ we have

$$\begin{array}{cc} I_{A}\left(0\right)I_{B}\left(0\right)I_{A}\left(0\right)\rho & =\mathrm{Tr}\left[A^{⊥}\rho \right]I_{A}\left(0\right)I_{B}\left(0\right)\left|01\rangle \langle 01\right|\\ & =\mathrm{Tr}\left[A^{⊥}\rho \right]\mathrm{Tr}[B^{⊥}|01\rangle \langle 01|]I_{A}\left(0\right)\left|10\rangle \langle 10\right|\\ & =0,\\ I_{A}\left(0\right)I_{B}\left(1\right)I_{A}\left(0\right)\rho & =\mathrm{Tr}\left[A^{⊥}\rho \right]I_{A}\left(0\right)I_{B}\left(1\right)\left|01\rangle \langle 01\right|\\ & =\mathrm{Tr}\left[A^{⊥}\rho \right]\mathrm{Tr}[B|01\rangle \langle 01|]I_{A}\left(0\right)\left|01\rangle \langle 01\right|\\ & =\mathrm{Tr}\left[A^{⊥}\rho \right]I_{A}\left(0\right)\left|01\rangle \langle 01\right|\\ & =\mathrm{Tr}\left[A^{⊥}\rho \right]\mathrm{Tr}[A^{⊥}|01\rangle \langle 01|]\left|01\rangle \langle 01\right|\\ & =\mathrm{Tr}\left[A^{⊥}\rho \right]|01\rangle \langle 01|,\\ I_{A}\left(1\right)I_{B}\left(0\right)I_{A}\left(1\right)\rho & =\mathrm{Tr}\left[A\rho \right]I_{A}\left(1\right)I_{B}\left(0\right)|10\rangle \langle 10|\\ & =\mathrm{Tr}\left[A\rho \right]\mathrm{Tr}[B^{⊥}|10\rangle \langle 10|]I_{A}\left(0\right)\left|10\rangle \langle 10\right|\\ & =\mathrm{Tr}\left[A\rho \right]I_{A}\left(1\right)|10\rangle \langle 10|\\ & =\mathrm{Tr}[A\rho ]\mathrm{Tr}[A|01\rangle \langle 01|]|10\rangle \langle 10|\\ & =\mathrm{Tr}[A\rho ]|01\rangle \langle 01|,\\ I_{A}\left(1\right)I_{B}\left(1\right)I_{A}\left(1\right)\rho & =\mathrm{Tr}\left[A\rho \right]I_{A}\left(1\right)I_{B}\left(1\right)|10\rangle \langle 10|\\ & =\mathrm{Tr}\left[A\rho \right]\mathrm{Tr}[B|10\rangle \langle 10|]I_{A}\left(1\right)\left|01\rangle \langle 01\right|\\ & =0. \end{array}$$

Consequently,

$$\begin{array}{cc} \sum_{x,y}\mathrm{Tr}\left[I_{A}\left(x\right)I_{B}\left(y\right)I_{A}\left(x\right)\rho \right] & =\mathrm{Tr}\left[A^{⊥}\rho \right]+\mathrm{Tr}\left[A\rho \right]\\ & =1. \end{array}$$

Thus, relation (iii) follows, and relation (iv) similarly follows.  □

**Theorem** **2.** *The instruments $I_{A}$ and $I_{B}$ have the Question Order Effect; namely, we have the following statements.*

*1.*  *For any $\psi =\sum_{\alpha ,\beta }c_{\alpha ,\beta }|\alpha ,\beta \rangle$ the relation*
  (32) $$\mathrm{Tr}[I_{B}\left(0\right)I_{A}\left(1\right)|\psi \rangle \langle \psi |]=\mathrm{Tr}[I_{A}\left(1\right)I_{B}\left(0\right)|\psi \rangle \langle \psi |]$$
  *holds if and only if ${\left|c_{00}\right|}^{2}={\left|c_{11}\right|}^{2}$.*
*2.*  *For any $\psi =\sum_{\alpha ,\beta }c_{\alpha ,\beta }|\alpha ,\beta \rangle$ the relation*
  (33) $$\mathrm{Tr}[I_{B}\left(1\right)I_{A}\left(0\right)|\psi \rangle \langle \psi |]=\mathrm{Tr}[I_{A}\left(0\right)I_{B}\left(1\right)|\psi \rangle \langle \psi |]$$
  *holds if and only if ${\left|c_{00}\right|}^{2}={\left|c_{11}\right|}^{2}$.*
*3.*  *Both Equations (32) and (33) hold only on a set of state vectors |ψ〉 with Lebesgue measure 0.*

**Proof.**  We have

$$\begin{array}{cc} \mathrm{Tr}[I_{B}\left(0\right)I_{A}\left(1\right)|\psi \rangle \langle \psi |] & =\mathrm{Tr}[A|\psi \rangle \langle \psi |]\mathrm{Tr}[I_{B}\left(0\right)|10\rangle \langle 10|]\\ & =\langle \psi |A|\psi \rangle \\ & ={\left|c_{10}\right|}^{2}+{\left|c_{11}\right|}^{2},\\ \mathrm{Tr}[I_{A}\left(1\right)I_{B}\left(0\right)|\psi \rangle \langle \psi |] & =\mathrm{Tr}[B^{⊥}|\psi \rangle \langle \psi |]\mathrm{Tr}[I_{A}\left(1\right)|10\rangle \langle 10|]\\ & =\langle \psi |B^{⊥}|\psi \rangle \\ & ={\left|c_{00}\right|}^{2}+{\left|c_{10}\right|}^{2}. \end{array}$$

Thus, assertion (i) holds.

$$\begin{array}{cc} \mathrm{Tr}[I_{B}\left(1\right)I_{A}\left(0\right)|\psi \rangle \langle \psi |] & =\mathrm{Tr}[A^{⊥}|\psi \rangle \langle \psi |]\mathrm{Tr}\left[I_{B}\left(1\right)\left|01\rangle \langle 01\right|\right]\\ & =\langle \psi |A^{⊥}|\psi \rangle \\ & ={\left|c_{00}\right|}^{2}+{\left|c_{01}\right|}^{2},\\ \mathrm{Tr}[I_{A}\left(0\right)I_{B}\left(1\right)|\psi \rangle \langle \psi |] & =\mathrm{Tr}[B|\psi \rangle \langle \psi |]\mathrm{Tr}[I_{A}\left(0\right)|10\rangle \langle 10|]\\ & =\langle \psi |B|\psi \rangle \\ & ={\left|c_{01}\right|}^{2}+{\left|c_{11}\right|}^{2}. \end{array}$$

Thus, assertion (ii) holds. Assertion (iii) easily follows from the above statements.  □

## 6. Topological Stability of QOE

If QOE holds for any state |ψ〉, and we considered only pure states, then it is stable if it holds in some small neighborhood $U_{\epsilon }(|\psi \rangle )$, in the Hilbert space metric.

**Theorem** **3.** *If QOE is valid for a state |ψ〉, then QOE is also valid in its small neighborhood.*

**Proof.**  By Theorem 2, the states for which QOE are not valid form a closed set, hence validating the QOE on an open set of states, so that the assertion follows.  □

Note that $I_{A}$ and $I_{B}$ can be represented as

$$\begin{array}{cc} I_{A}\left(0\right)\rho & =|01\rangle \langle 00|\rho |00\rangle \langle 01|+|01\rangle \langle 01|\rho |01\rangle \langle 01|.\\ I_{A}\left(1\right)\rho & =|10\rangle \langle 10|\rho |10\rangle \langle 10|+|10\rangle \langle 11|\rho |11\rangle \langle 10|.\\ I_{B}\left(0\right)\rho & =|10\rangle \langle 00|\rho |00\rangle \langle 10|+|10\rangle \langle 10|\rho |10\rangle \langle 10|.\\ I_{B}\left(1\right)\rho & =|01\rangle \langle 01|\rho |01\rangle \langle 01|+|01\rangle \langle 11|\rho |11\rangle \langle 01|. \end{array}$$

They are not atomic in the sense of Equation (8): If $I_{A}\left(1\right)\rho =V\rho V^{†}$, then $M=V^{†}V$. Since the range of $V\rho V^{†}$ is spanned by |01〉, *V* is of rank one, and so is $M=V^{†}V$. This is contradiction, since A=M, i.e., Tr[Aρ]=Tr[Mρ] for all ρ, and A=|1〉〈1|⊗I is of rank two.

## 7. Conclusions

We have shown that quantum formalism is consistent with the combination of two fundamental psychological effects: QOE and RRE. Thus, the objection presented in [24] and challenged applications of this formalism to psychology was eliminated.

This is the good place to make the following general remark. This area of research, quantum-like modeling, is quite young and it is not so well established. Researchers outside this small community can be curious whether borrowing quantum probability formalism from physics would not lead to paradoxes similar to the paradoxes generated by using the classical probability formalism in decision-making.

Before this paper, the situation was really alarming, although this alarm-signal was generally ignored by the majority of newcomers to quantum-like modeling, and the experts well understood that the project of quantum decision-making can collapse.) The authors of [24] showed that the straightforward application of quantum formalism, i.e., the attempt to proceed with representation of observables by Hermitian operators is incompatible with some natural constraints generated by human psychological behavior. The first attempt to appeal to more general quantum models with atomic instruments was not successful [79], the same no-go result as in [24]. In the present paper, this problem was resolved by using non-atomic quantum instruments. Thus, the quantum psychology project was saved (well, maybe just for a while...). Finally, we remark that the use of non-atomic instruments is not so common in genuine quantum physics. An appeal to them was really counter-intuitive, and the positive solution of the problem was unexpected.

Thus, to combine QOE and RRE, one has to consider the general quantum measurement theory based on quantum non-atomic instruments, the operational approach [65,69]. It is impossible to proceed with measurements of the projection type. The essence of the operational approach is treatment of systems not as isolated, but as interacting with an environment (theory of open quantum systems). In psychology, this is “mental environment” (cf. [35]).

Of course, even by resolving the concrete problem of quantum-like modeling of QOE and RRE, we should be cautious by using quantum-like modeling in psychology. There are plenty of other psychological effects. One has to be sure that their combination is covered by theory of quantum instruments. One such effect is based on so-called QQ-equality (QQE), which was derived by Busemeyer et. al. [80] and checked for a variety of opinion polls. The quantum-like model constructed in this paper can be extended (via extension of the state space) to a more general model covering combination of QQE, RRE, and QQE. However, such a model becomes very technically complicated. Therefore, we prefer to concentrate on the presentation of the simpler model covering QQE and RRE. In psychology, and generally outside of physics, theory of quantum instruments has not yet been used, with the exception of [35]. It is better to proceed slowly, without complex technicalities which would shadow the essence of the operational approach.
